# Aberrant Expression of p63 in an Adenocarcinoma of the Prostate That Has Metastasized to the Oral Cavity

**DOI:** 10.7759/cureus.22753

**Published:** 2022-03-01

**Authors:** Rafiq Khalid, Anand Ramanathan, Heng Tee Lun, Daniel Lim

**Affiliations:** 1 Department of Oral and Maxillofacial Clinical Sciences, Faculty of Dentistry, Universiti Malaya, Kuala Lumpur, MYS; 2 Department of Oral Maxillofacial Surgery and Oral Diagnosis, Kulliyyah of Dentistry, International Islamic University Malaysia, Kuantan, MYS; 3 Oral Cancer Research and Coordinating Centre, Faculty of Dentistry, Universiti Malaya, Kuala Lumpur, MYS

**Keywords:** p63, adenocarcinoma, prostate cancer, jaw bone, metastasis

## Abstract

Metastasis specifically to the oral cavity is uncommon in cases of prostate adenocarcinoma. Only 4% of prostate cancer patients present with metastases to the oral cavity originating from the prostate. In the oral cavity, the mandible is the primary site of metastases. p63 is said to be a reliable marker to distinguish benign from malignant lesions of prostate origin, with benign lesions staining positive and malignant lesions staining negative. However, in rare instances, malignant prostate lesions have shown aberrant expression of p63. This case report highlights such a rare incidence of metastasis of prostate adenocarcinoma to the oral cavity involving the right buccal mucosa and the right side of the mandible and having an aberrant expression of p63 in a 76-year-old Chinese male. A computed tomography (CT) scan and bone scan revealed multiple bone metastases, and in three months, the patient succumbed to the disease. Thus, p63 is not exclusively expressed in benign lesions of the prostate, as the aberrant expression may also be evident in malignant lesions such as prostate adenocarcinoma. Therefore, the determination of benign or malignant lesions of the prostate using p63 must be interpreted with caution.

## Introduction

Metastasis to the jaw accounts for 1-2% of oral malignancies, with the mandible being the most common location for jaw bone metastasis, mainly occurring in the molar region [1]. Almost 75% of patients with advanced prostate cancer present with distant metastasis, mainly to the lumbar spine, ribs, and pelvis [2]. However, metastasis to the oral cavity, however, is uncommon in prostate cancer, with only 4% of patients having oral metastases originating from the prostate [1].

Patients with oral metastases commonly present clinically with fast-growing swelling, pain, and numbness. It is generally observed that the prognosis of prostate cancer with distant metastasis is unfavourable [1,3].

P63 is a reliable marker to distinguish benign from malignant lesions of prostate origin, with benign lesions staining positive and malignant lesions such as prostate adenocarcinoma staining negative. However, it has been documented that there are rare instances, where malignant prostate lesions have shown aberrant staining with p63 [4]. This case report highlights a rare incidence of metastasis of prostate adenocarcinoma to the oral cavity and has aberrant staining with p63.

## Case presentation

**Figure 1:**
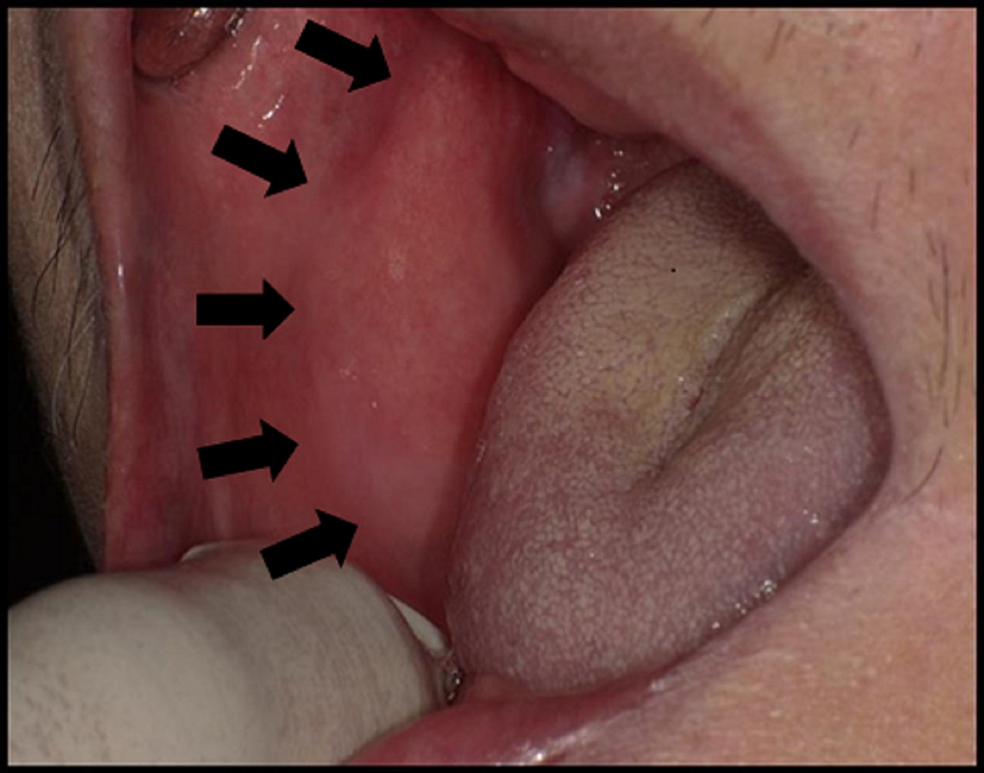
Intraoral photograph showing smooth, well-defined, firm mass over the right buccal mucosa with normal overlying mucosa.

A 76-year-old Chinese man came to the clinic with a complaint of swelling over the right buccal mucosa with on and off pain and numbness at his right chin in December 2017. The symptoms started two months prior. Clinically, his buccal mucosa was smooth, and a well-defined firm mass could be palpable over the normal overlying buccal mucosa (Figure 1). He was completely edentulous with no restriction of mouth opening.

The patient was a known case of end-stage prostate cancer. He was first diagnosed with adenocarcinoma of the prostate in June 2014 via transurethral resection of the prostate (TURP). The Gleason score was 4+5 = 9, and his prostate-specific antigen (PSA) was high (65 ng/mL). A bone scan in July 2014 revealed multiple bone metastases involving the left 4th rib, 4th lumbar of the spine, sacrum, and left pubic bone but not involving the mandible. He was treated with androgen deprivation therapy (Lucrin) until December 2015, followed by seven months of antineoplastic agents (Abiraterone acetate) from December 2016 to July 2017 due to a persistent high baseline of PSA value >100 ng/mL. The disease progressed and the patient was treated with nonsteroidal antiandrogen (Enzalutamide) from July 2017. His PSA value did not respond to the therapy. By October 2017, his PSA was 120 ng/mL, which coincided with his first clinical symptom of numb chin syndrome.

**Figure 2:**
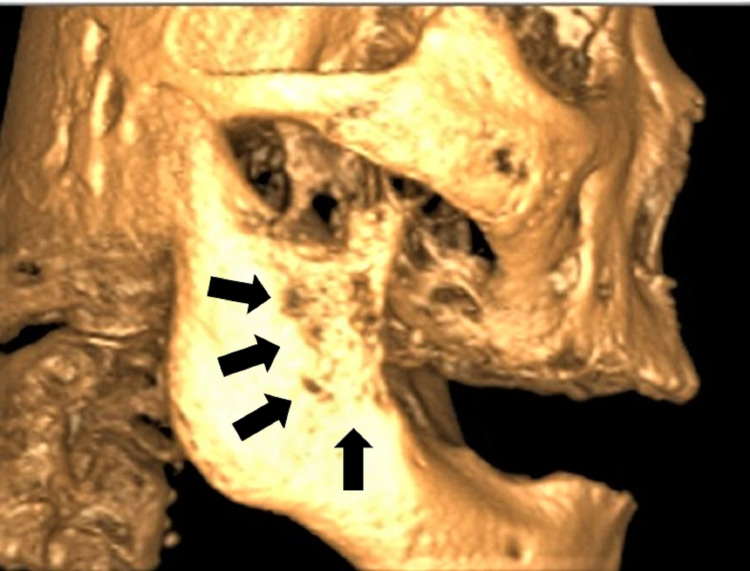
CBCT image showing erosion of bone at the anterior part of right ramus of mandible and retromolar region.

A cone-beam computed tomography (CBCT) showed erosion of bone in the anterior part of the ascending ramus and retromolar region (Figure 2). An incisional biopsy was then done on the lesion.

**Figure 3:**
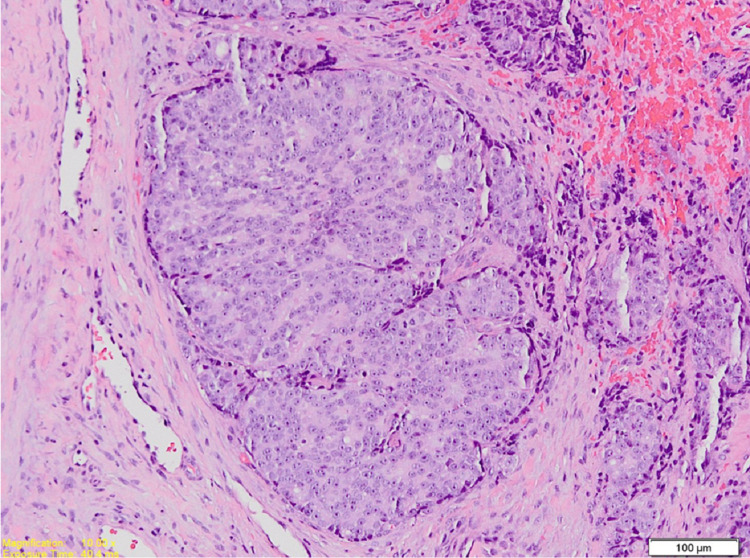
Photomicrograph shows a tumour island consisting of central round cells and peripheral spindle cells (magnification ×100, stain H&E).

**Figure 4:**
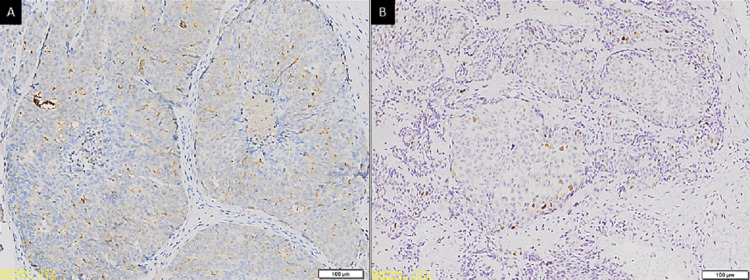
Photomicrographs showing (A) immunopositive cytoplasmic staining of tumour cells with PSA (magnification ×100) and (B) scattered immunopositive staining of tumour cells with p63 (magnification ×100).

The haematoxylin and eosin (H&E) slides of the lesion demonstrated tumour islands consisting of central cells with hypochromatic and vacuolated nuclei, pale eosinophilic cytoplasm, and exhibited pleomorphism. The peripheral basal tumour cells were spindle-shaped and appeared hyperchromatic (Figure 3), with areas of extensive comedonecrosis seen. The central tumour cells were immunopositive for PSA (Figure 4A) and cytokeratin (CK; weak). The peripheral basal tumour cells were immunopositive for PSA, CK (weak), and p63 (scattered) (Figure 4B). Both the central tumour cells and the basal tumour cells were negative for CK7 and CK20. Considering the H&E and immunohistochemistry presentation of the case, a diagnosis of adenocarcinoma metastasis from the prostate was reported.

**Figure 5:**
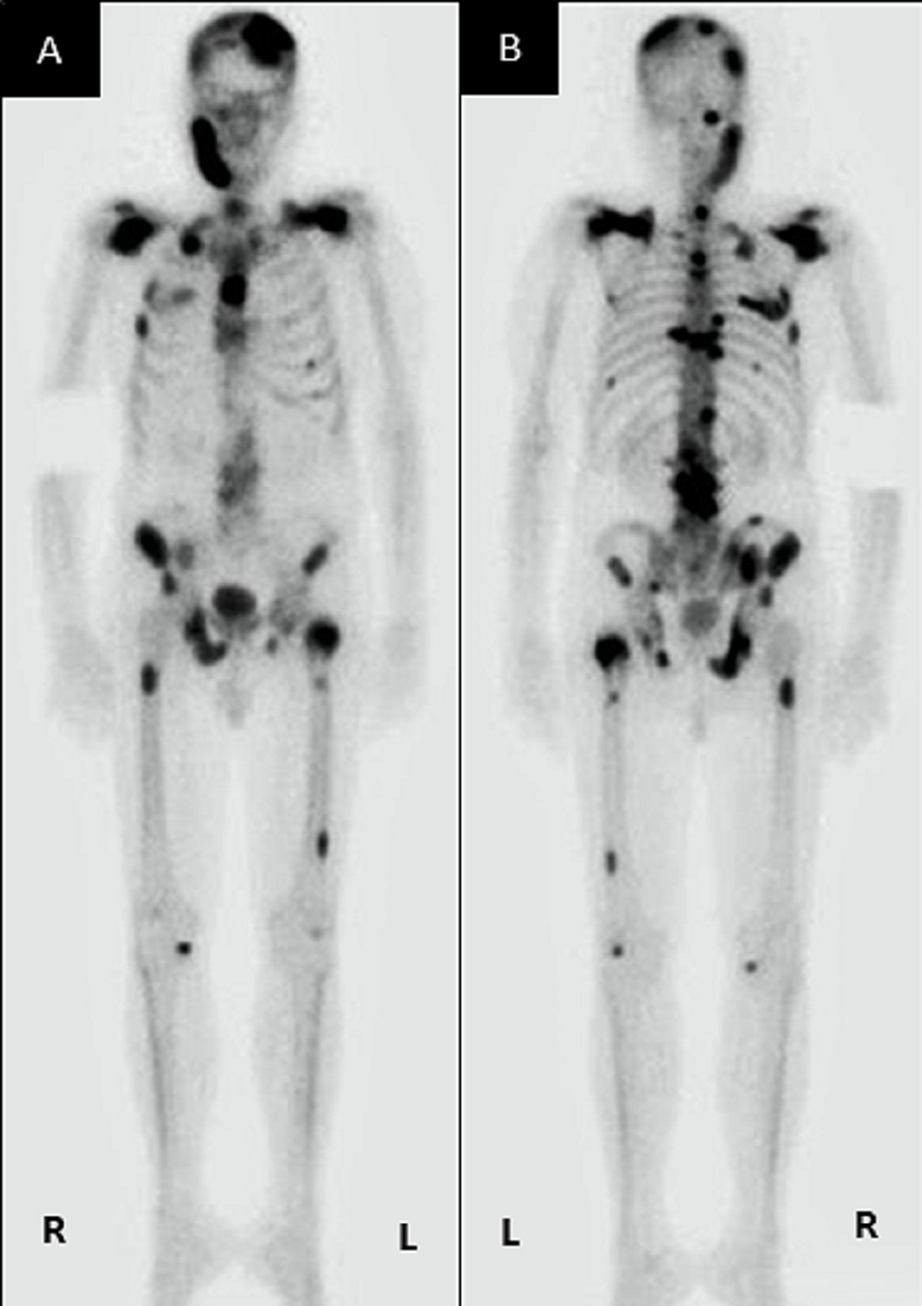
Bone scan images (A) anteroposterior view and (B) posteroanterior view showing multiple distant bone metastases to the mandible, sternum, ribs, ilium, femur and vertebrae.

The attending oncologist was informed of the metastatic finding. Following this, a computed tomography (CT) scan and bone scan (Figure 5A, 5B) were done for this patient, revealing multiple bone metastases to the sternum, ribs, ilium, femur, and vertebrae as well as the mandible. Subsequently, the patient was placed on chemotherapy treatment. However, three months later, unfortunately, the patient succumbed to the disease and died.

## Discussion

The ability to metastasize is one of the inherent properties of malignancies. This process is somewhat regulated to be site-specific. The "seed and soil" hypothesis mentions that the metastatic tumour (seed) will only grow in an organ that provides a suitable environment (soil). The metastatic process is complex; the tumour cells need to be detached from the primary tumour and invade adjacent tissues and enter the vascular or lymphatic vessels before lodging at a distant site. These tumour cells then survive by angiogenesis, tumour dormancy, and evading apoptosis [1]. The distant spread of tumour from the prostate to the maxillofacial region may occur via Batson’s plexus. During a transient increase in intraabdominal pressure, this route allows the retrograde spread of tumours via the valveless prevertebral veins [5]. This is evident in the bone scan of this patient, showing metastasis along the vertebra as well as to the maxillofacial region and skull.

In this case, the patient was a known case of end-stage prostate cancer, as in cases reported previously in the literature [6-8]. The time interval between the diagnosis of prostate cancer and the occurrence of metastasis to the mandible ranges from 2 months [8] to 120 months [6]. It was 45 months in the present case. However, in many other cases, oral lesions were seen as the first lesions to manifest before the diagnosis of prostate cancer [9-11], thus helping to find the primary cancer. 

Clinically, oral metastatic lesions from the prostate present as a fast-growing swelling with pain and/or paraesthesia (numbness) in the chin area (numb chin syndrome) if the mandible is involved [1]. In the present case, the patient reported swelling, pain, and paraesthesia (numb chin syndrome) in the right chin region, thus suggesting the involvement of the mandible. However, the swelling was also present in the right buccal mucosa, indicating that the lesion had involved not only the mandible but also the oral soft tissue, similar to a case reported by Mohamed and Suleiman [8]. The other clinical features that have been reported in oral metastasis of prostate cancer are trismus or limited mouth opening [7] and necrotic bone due to medication-induced osteonecrosis [12].

The molar region of the mandible has been reported as the most commonly affected hard tissue site in the maxillofacial skeleton, and the attached gingiva (only two cases) was the most commonly involved soft tissue site for oral metastases from the prostate [1]. A review reported that the most commonly affected sites for metastasis of the mandible were the molar and premolar regions, followed by the ascending ramus, angle of the mandible, and mental region [13]. In this case, the metastases were to the anterior part of the ascending ramus and the retromolar region, as seen in the CBCT and extending into the oral soft tissue of the right buccal mucosa. Most of the cases of prostate metastasis to the mandible had osteolytic lesions [10] such as in this case, but some cases have reported osteoblastic lesions with a sunburst [9,11] appearance as seen in osteosarcoma.

Histopathogically, the use of CK7 and CK20 markers in tandem may be utilised to aid in the diagnosis of prostate carcinoma. A negative stain for both CK7 and CK20 would suggest prostate carcinoma [14] and has been reported in many cases [9,15]. A study concluded that the PSA marker, which is highly sensitive in prostate cancer, can be used to differentiate prostate carcinoma from other forms of carcinoma [16]. In this case, the utilisation of CK was used to verify a neoplasm of epithelial origin. With regards to the case under discussion, both CK7 and CK20 were negative, whereas PSA was strongly positive, indicating prostate adenocarcinoma.

In previous studies, the utilisation of high molecular weight cytokeratin (HMWCK) markers has also been demonstrated to allow differentiation between prostate carcinoma from benign prostate lesions, whereby there is a loss of staining of the basal cells in cases of prostate carcinoma [17]. The use of HMWCK would therefore also be a helpful option to confirm a diagnosis of prostate adenocarcinoma. Additionally, alpha-methylacyl-CoA racemase (AMACR) may also be utilised as a viable option to diagnose prostate adenocarcinoma [15]. AMACR is a carcinoma stem cell marker that has emerged as a prostate cancer marker [18] but is also highly expressed in other cancers such as renal cancer, liver cancer, and colon cancers [19].

It has been shown over the years that p63 is a reliable marker to distinguish benign from malignant lesions of prostate origin, with benign lesions staining positive and malignant lesions such as prostate adenocarcinoma staining negative [4,9]. However, it has also been documented that there are instances, although uncommon, where even malignant prostate lesions have shown aberrant staining with p63 [20,21], such as in this case.

The use of p63 must also be interpreted with caution in lesions that occur within the oral cavity, as certain salivary gland tumours such as basal cell adenocarcinoma, polymorphous adenocarcinoma, and adenoid cystic carcinoma tend to show immunopositive to p63, which is a known myoepithelial marker. These salivary gland tumours also often have ductal structures similar in pattern to those seen in prostate adenocarcinoma. However, in this case, the use of PSA excludes a salivary gland tumour. It is also worth noting that comedonecrosis, as reported in this case, has also been associated with high-grade disease [22], explaining in part the aggressiveness of the disease in this patient.

Metastasis of prostate cancer to the mandible is considered to occur at an advanced stage and the prognosis is poor, with survival being as low as three weeks [9]. In the present case, the patient died three months after the oral metastasis.

## Conclusions

In conclusion, p63 is not exclusively expressed in benign lesions of the prostate, as the aberrant expression may also be evident in malignant lesions such as prostate adenocarcinoma. Therefore, the determination of benign or malignant lesions of the prostate using only p63 must be interpreted with caution. The expression of proteins demonstrated through immunohistochemistry should be used as an adjunct diagnostic tool in the context of the presence or absence of histopathological features in a tumour.
